# Efforts to Downsize Base Editors for Clinical Applications

**DOI:** 10.3390/ijms26052357

**Published:** 2025-03-06

**Authors:** Beomjong Song

**Affiliations:** Department of Anatomy, College of Medicine, Soonchunhyang University, Cheonan 31151, Republic of Korea; beomjong.song@sch.ac.kr

**Keywords:** CRISPR, gene editing, base editor, AAV

## Abstract

Since the advent of the clustered regularly interspaced short palindromic repeats (CRISPR) system in the gene editing field, diverse CRISPR-based gene editing tools have been developed for treating genetic diseases. Of these, base editors (BEs) are promising because they can carry out precise gene editing at single-nucleotide resolution without inducing DNA double-strand breaks (DSBs), which pose significant risks of genomic instability. Despite their outstanding advantages, the clinical application of BEs remains challenging due to their large size, which limits their efficient delivery, particularly in adeno-associated virus (AAV)-based systems. To address this issue, various strategies have been explored to reduce the size of BEs. These approaches include truncating the nonessential domains and replacing the bulky components with smaller substitutes without compromising the editing efficiency. In this review, we highlight the importance of downsizing BEs for therapeutic applications and introduce recent advances in size-reduction strategies. Additionally, we introduce the ongoing efforts to overcome other limitations of BEs, providing insights into their potential for improving in vivo gene editing.

## 1. Introduction

The development of clustered regularly interspaced short palindromic repeats (CRISPR) technology has ushered in a new era in the field of gene editing. The RNA-dependent mechanism of DNA targeting confers a distinct advantage to the CRISPR system over other gene editing technologies, such as zinc-finger nucleases and transcription activator-like effector nucleases, which require protein engineering for DNA targeting [1,2,3]. However, gene editing using the CRISPR system is not trouble-free compared with other gene editing tools. Conventional gene editing is based on the induction of DNA double-strand breaks (DSBs). The DSBs are then repaired by cellular DNA repair mechanisms, such as nonhomologous end joining (NHEJ) or homology-directed repair (HDR), with each pathway having its limitations [4]. Although NHEJ is a dominant pathway, it is very error-prone, which results in the generation of small insertions and deletions (indels). In contrast, HDR is an accurate pathway, but limited to specific cell cycle phases (S and G2), and its efficiency is low. Moreover, DSBs can lead to a p53-mediated DNA damage response, undesired large chromosomal deletions, or genomic rearrangement [5,6,7]. These shortcomings are fatal in the context of clinical applications of gene editing tools, which require a technology that is free from DSB generation.

**Figure 1:**
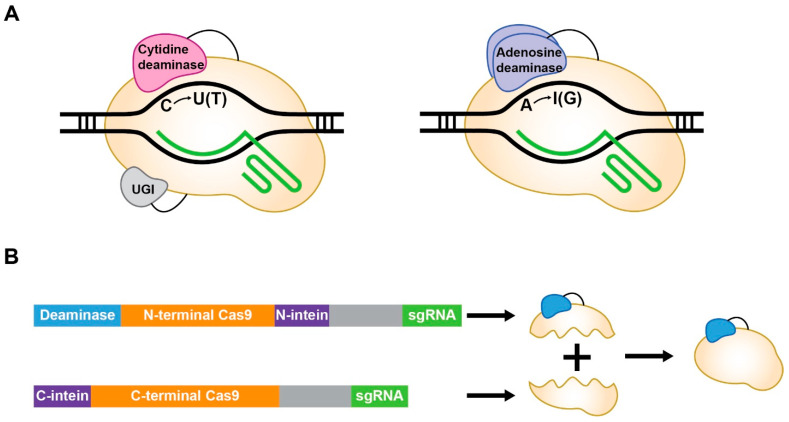
Structure of a BE. (**A**) Schematic diagram of a cytosine and an adenine base editor. (**B**) Schematic diagram of a dual AAV system.

A base editor (BE) is a novel gene editing tool that is independent of DSBs [8,9]. BEs are composed of a CRISPR-associated (Cas) protein and a deaminase and are referred to as cytosine BE (CBE) and adenine BE (ABE), depending on the type of deaminase (Figure 1A). In the initial version of the CBE (BE1), Cas9 from *Streptococcus pyogenes* (SpCas9) was functionally inactivated (dead Cas9, dCas9) to avoid DSB generation [8]. Subsequently, the function of dCas9 was partially restored (nickase Cas9, nCas9) for better editing efficiency (BE3). The editing efficiency of BE3 was further improved by adding a uracil glycosylase inhibitor (UGI), codon optimization, and ancestral reconstruction to generate BE4, BE4max, and AncBE4max, respectively [10,11,12]. As cytidine deaminases, rat apolipoprotein B-editing catalytic subunit 1 (rAPOBEC1), human APOBEC3A (hA3A), and activation-induced cytidine deaminase (AID) have been used [8,13,14]. Similarly, an adenosine deaminase, tRNA adenosine deaminase (TadA), has been fused to nCas9 to generate an ABE [9]. Because natural TadA is ineffective on DNA, mutated TadA (TadA*) is used in ABEs. Through several attempts, including additional mutations to TadA* (TadA-8e) and codon optimization, ABEs with high editing efficiency, such as ABE7.10, ABEmax, and ABE8e, have been developed [9,12,15]. CBEs and ABEs convert cytosine and adenine to uracil and inosine, respectively. The resulting U-G and I-T mismatches are repaired by cellular mechanisms, and new T-A and G-C pairs are generated. Consequently, BEs can accomplish C-to-T or A-to-G editing in a target site at single-nucleotide resolution.

In addition to these properties, the delivery of BEs is also an important factor that can affect the efficiency of gene editing for therapeutic purposes. In general, the adeno-associated virus (AAV) vector is considered the most promising delivery platform because it can efficiently infect cells persistently and exhibits widespread tissue tropism [16,17]. It has been proven to be safe [18], and clinical applications of two AAV vectors have been approved by the US Food and Drug Administration [19,20]. Nevertheless, AAV vectors and BEs do not work well together. The loading capacity of AAV vectors is approximately 4.7 kb [21], whereas the size of BEs ranges from 4.2 to 5.2 kb; thus, they cannot be loaded into a single AAV vector. To overcome this problem, BEs may be split and loaded into dual AAV vectors (Figure 1B) [22,23]. However, a dual AAV system exhibits reduced transduction efficacy, thereby lowering the gene editing efficiency [24]. Although the low efficiency of a dual AAV system can be improved with a high-dose AAV, it can result in liver toxicity [25].

To address this issue, there have been various attempts to reduce the size of BEs without compromising their gene editing efficiency. In many studies, small-sized variants of Cas proteins and deaminases have been reported, and hypercompact BEs have been generated using these components. In addition, deleting unnecessary protein domains represents another way to reduce the size of BEs.

In this review, we summarize the various efforts to develop BEs that are small enough to be loaded into a single AAV vector. We also present the features of each hypercompact BE and discuss its prospects.

## 2. Truncation of the BE Components

**Figure 2:**
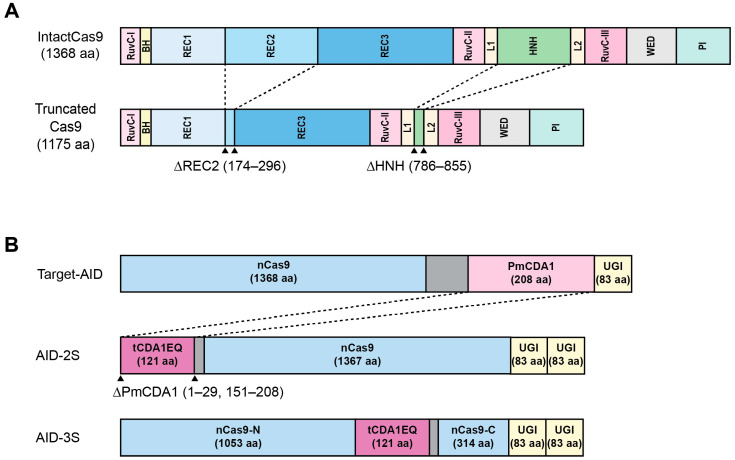
The truncation of the BE components. (**A**) The truncation of the REC2 and HNH domains can reduce the size of the Cas9 protein. (**B**) The truncation of PmCDA1 generates a small-sized PmCDA1.

A structural study revealed that single deletions of the REC2, REC3, HNH, and RuvC-III domains do not impair the DNA binding affinity of dCas9 [26]. Each domain deletion can be stacked to generate small-sized dCas9, although it is less functional compared with the single-deletion variants. A similar strategy was applied to a BE to reduce its size [27]. For ABE8.17 [28], the deletion of REC2 (Δ174–296), REC3 (Δ509–672), HNH (Δ786–855), or RuvC-III (Δ1044–1081) was evaluated [27], and ABE8.17 tolerated a single deletion of REC2 and HNH. In addition, ABE8.17 with a double deletion (REC2 + HNH) retained an editing efficiency similar to that of ABE7.10 (Figure 2A). Notably, for AncBE4max carrying an REC2 or an HNH deletion, the editing efficiency was significantly lower.

Based on a previous study reporting that ABE8e outperforms ABE8.17 [29], the double deletion was introduced into ABE8e and the resulting sABE exhibited various editing efficiencies (10.3–43%) in HEK293T cells depending on the target site [27]. Although the editing efficiency of sABE is inferior to that of ABE8e, the off-target effect is much lower in sABE. The size of sABE is 4.15 kb, which is small enough to be loaded into a single AAV vector. However, sABE delivered to the liver of mice via an AAV vector resulted in low editing efficiency (1.09%), suggesting that further optimization is required.

Alternatively, deaminase can be truncated to reduce the BE size (Figure 2B). DNA deaminases have an intrinsic ability to bind to DNA, which causes nonspecific deamination. Therefore, deleting the DNA-binding domain (DBD) can reduce not only the size of the BE, but also the off-target effects. *Petromyzon marinus* cytidine deaminase (PmCDA1) is a component of CBE known as target activation-induced cytidine deaminase (Target-AID) [14]. A structural analysis predicted that the DBD moieties are located at both the C- and N-terminal ends [30]. A deletion in the C-terminal end (Δ162–208) reduced the editing activity of PmCDA1 in the absence of UGI [31]. The editing efficiency of the truncated PmCDA1 (tCDA1) was partially restored by an additional truncation at the C-terminal end (Δ151–161) and the N-terminal end (Δ1–29). The editing efficiency was further improved by introducing mutations (W122E and W139Q) into PmCDA1 (30–150) (tCDA1EQ), and the off-target effects were significantly reduced by adding UGI. Notably, fusing tCDA1EQ to the N-terminal of nCas9 (AID-2S) showed various editing efficiencies depending on the target sites, whereas inlaying tCDA1EQ within nCas9 (AID-3S) resulted in consistent activity with a wider editing window. Just as Target-AID has few RNA off-target effects [31], AID-2S and AID-3S did not show detectable off-target effects on RNA [30]. The size of AID-2S and AID-3S was compacted by replacing nCas9 with nSaCas9. The resulting SaAID-2S and SaAID-3S exhibited an editing efficiency comparable to that of Target-AID harboring SaCas9, with no detectable RNA off-target effects. In particular, the off-target effect on the DNA was significantly reduced in SaAID-3S. The size of SaAID-2S and SaAID-3S was small enough to be loaded into a single AAV vector; however, only SaAID-2S exhibited significant editing efficiency in HEK293T cells when tested via AAV vectors.

## 3. Adoption of Small Cas9 Orthologs

**Figure 3:**
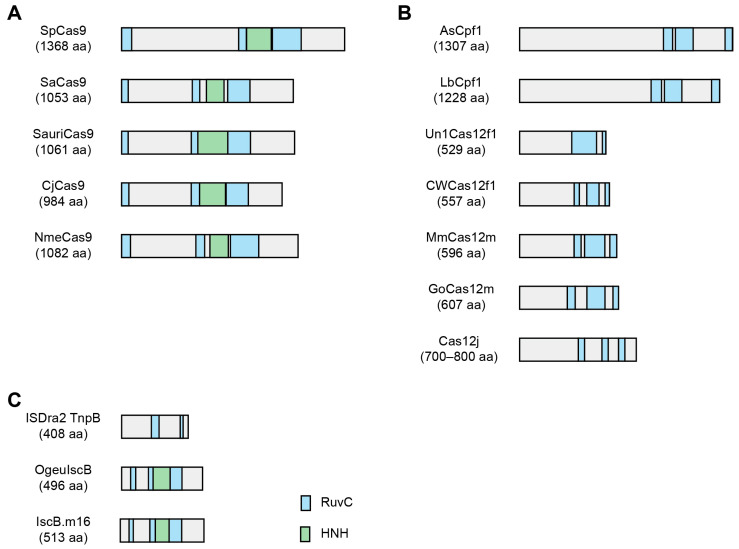
Diverse hypercompact CRISPR and OMEGA systems. (**A**) Small-sized Cas9 orthologs. (**B**) Hypercompact Cas12 orthologs. (**C**) Small-sized ancestors of Cas9 and Cas12.

Since the CRISPR system was first discovered [32], diverse Cas9 protein orthologs have been identified through phylogenetic studies [33]. For some Cas9 orthologs, the REC domain is intrinsically truncated [34,35] and has a relatively smaller size compared with SpCas9; thus, they may be useful for constructing hypercompact BEs (Figure 3A).

Cas9 from *Staphylococcus aureus* (SaCas9) consists of 1053 amino acids [36]. Different from SpCas9, SaCas9 recognizes NNGRRT as a protospacer adjacent motif (PAM) [36,37] and cleaves target sites with a high efficiency and specificity compared with SpCas9 [38].

For generating small-sized BEs, SaCas9 could replace nCas9 in BE3 (SaBE3) [39]. SaBE3 exhibited efficient editing (50–75%), and a variant of SaBE3 adopting Sa-KKH that recognizes NNNRRT PAM (SaBE3-KKH) expanded the targetable sites. Similarly, nSaCas9-KKH was adopted in ABE7.10 and ABE8e [15,40]. The SaKKH-ABE exhibited efficient editing of HEK293T cells and mouse embryos with no detectable off-target effects. ABEmax variants adopting SaCas9 and SaCas9-KKH also exhibited efficient activity with a targeting scope different from that of ABEmax [41]. The size of ABEmax may be reduced by removing the wild-type TadA domain (miniABEmax) [42]. In addition, the RNA off-target effect was improved by introducing a mutation (V82G) into TadA*. To further downsize the construct, nCas9 in the miniABEmax (V82G) was replaced with SaCas9 nickase (nSaCas9) [43]. Furthermore, shifting the base editing domain to within nSaCas9 improved the editing efficiency. The small ABE, designated microABEI744, exhibited lower RNA off-target effects compared with the SaCas9-based miniABEmax (V82G) and SaCas9-based ABEmax, whereas there was no improvement in the DNA off-target effects. As expected by its small size, microABEI744 could be packaged into a single AAV vector; however, the transduction of the AAV carrying microABEI744 to HEK293A cells exhibited very low editing efficiency (0.24%).

Cas9 from *Staphylococcus auricularis* (SauriCas9) has a small size (1061 amino acids), comparable to that of SaCas9 [44]. Although SauriCas9 is genetically similar to SaCas9, it recognizes a simpler PAM sequence (NNGG), and exhibits reduced off-target effects. SauriCa9 nickase was adopted in SauriBE4max, SauriABEmax, and SauriABE8e, and those small BEs showed various editing efficiencies of HEK293T cells depending on the target site [45].

Another Cas9 ortholog from *Campylobacter jejuni* (CjCas9) is composed of 984 amino acids [46] and recognizes N_4_ACAC, N_4_RYAC (R = A or G), and N_4_ACA PAMs [46,47]. CjCas9 was optimized with 22 nt gRNA and its activity was comparable to that of SaCas9, whereas CjCas9 exhibited higher specificity [46]. CjABE was based on ABE7.10 and nCas9 was replaced with CjCas9 nickase (nCjCas9) [48]. The application of CjABE using AAV vectors into cells with a C-to-T mutation in *the TERT* promoter region resulted in a high correction rate (approximately 97%). The same AAV vectors were effective in a mice model carrying the same mutation, although unwanted editing of A positioned next to the target A occurred (bystander mutation). In some studies, CjCas9 exhibited relatively low activity with specific PAM sequences (N_3_RGCAC and N_3_CGYAC) (Y = C or T) [46,49,50]. This bias was eliminated by engineering CjCas9 (L58Y/D900K) (enCjCas9) [50]. enCjCas9 nickase was adopted in the Target-AID, and the enCjCas9-AID induced C-to-T editing at the target site, whereas the CjCas9-AID did not show any activity. However, the editing efficiency of the enCjCas9-AID was lower compared with that of Target-AID. In another study, CjCas9 nickase was combined with rAPOBEC1 from BE4max and evolved TadA from ABE8e to generate cjCBEmax and cjABE8e, respectively [45,51]. cjCBEmax and cjABE8e exhibited editing efficiencies of up to 13.5% and 9.4%, respectively. Both CjCas9-based BEs were further improved by adopting enCjCas9. The administration of cjABE8e using single AAV vectors into HEK293T cells resulted in 24.0–91.9% editing efficiency, without detectable off-target mutations.

Cas9 from *Neisseria meningitidis* (NmeCas9 or NmCas9) consists of 1082 amino acids and is subdivided into Nme1Cas9, Nme2Cas9, and Nme3Cas9 [52,53]. NmeCas9 has low off-target effects because of its relatively longer PAM sequences (N_4_GATT for Nme1Cas9, N_4_CC for Nme2Cas9, and N_4_CAAA for Nme3Cas9) and guide sequence (24 nt) [54]. Consistently, nNme2-CBE (BE4max variant including Nme2Cas9 nickase) exhibited less Cas9-dependent DNA off-target effects while retaining its on-target editing activity, which was comparable to that of BE4max [55]. Notably, nNme2-CBE was less effective at target sites with a GC context. The inefficiency in a GC context was improved using evolved APOBEC1 (eA1), evolved CDA1 (eCDA1), and hAPOBEC3A, instead of the original rAPOBEC1 [55,56,57,58].

Nme2-ABE8e (ABE8e adopting Nme2Cas9 nickase) showed robust activity with low off-target effects [45,59]. The size of Nme2-ABE8e (4998 bp) is close to the packaging limit of the AAV vector; however, it could be further reduced (4860 bp) by replacing the U6 promoter with the mini6 promoter. Although the replacement of the promoter compromised the editing efficiency in vivo (6.59% with U6 vs. 1.62% with miniU6 in the *FAH* gene), correcting a point mutation in the *FAH* gene with either Nme2-ABE8e-U6 or Nme2-ABE8e-miniU6 restored the phenotype in FAH-deficient mice.

A Cas9 ortholog from *Neisseria cinerea* (NcCas9) is closely related to NmeCas9 and has the same size (1082 amino acids) [60]. Because NcCas9 recognizes N_4_GYAT (N_4_GTAT > N_4_GCAT) PAM most efficiently and N_4_ATAT/N_4_GTTT suboptimally, it can target DNA sequences in various contexts. Combining NcCas9 nickase (nNcCas9) and enhanced AID (eAID) with hyperactive mutations [61] resulted in nNc-CBE, which exhibited an editing efficiency ranging from 7.0% to 46.7% [60]. Similarly, nNc-ABE composed of nNcCas9 and ABE8e showed A-to-G editing efficiencies from 7.0% to 46.3% for various target sites in HEK293T cells.

## 4. Employment of Cas12f (Also Known as Cas14) Orthologs

The CRISPR-Cas12 system encompasses 10 subclasses (from 12a to 12m), in which the sizes range from approximately 500 to 1300 amino acids (Figure 3B) [62,63]. In particular, Cas12f from uncultivated archaea (Un1Cas12f1) is the smallest (529 amino acids). Although the small size of Un1Cas12f1 is attractive for developing hypercompact BEs, there are several limitations. Un1Cas12f1 interacts with only single-stranded DNA (ssDNA) [64]. In addition, Un1Cas12f1 is less active in eukaryotic cells compared with prokaryotic cells [65,66]. In a recent study, Un1Cas12f1 interacted with double-stranded DNA (dsDNA) when a PAM sequence was close to the target site [65]. The inertness of Un1Cas12f1 in eukaryotic cells was also solved by redesigning the gRNA and engineering the Un1Cas12f1 protein [67]. To this end, the gRNA scaffold was modified by changing the sequence, and mutations (D143R/T147R/K330R/E528R) were introduced into Un1Cas12f1 to increase DNA-binding activity. Moreover, the DNA cleavage function of the mutated Un1Cas12f1 was abolished by additional mutations (D326A/D510A). The resulting engineered Un1Cas12f1, designated dCasMINI, recognized TTTR PAM. To verify its performance, dCasMINI was fused to VP64-P65AD-Rta (VPR), which has been used for gene expression [68], and dCasMINI exhibited DNA-binding activity comparable to that of dCas12a from *Lachnospiraceae bacterium* (LbCas12a, also known as LbCpf1) in human cells [67]. Consistently, dCasMINI-ABE, a complex of dCasMINI and TadA-TadA-8e heterodimer, performs A-to-G editing with an efficiency similar to that of Cas12a-based ABE.

In another study, the activity enhancement of Un1Cas12f1 was achieved just by remodeling the gRNA [66]. In this study, multiple sites on the gRNA were modified (ge4.1), which synergistically improved the activity of Un1Cas12f1. The enhanced activity and off-target effects of Un1Cas12f1 were comparable to those of SpCas9 and Cas12a from *Acidaminococcus* sp. (AsCas12a, also known as AsCpf1), respectively. With ge4.1, diverse miniABEs were developed [69]. The miniABEs varied in their mutations in dUn1Cas12f1 and TadA*, and in the number (monomer vs. dimer) and position of TadA*. Each miniABE variant exhibited different editing scopes and off-target effects on the DNA and RNA. The combination of Un1Cas12f1 and ge4.1 was also used to produce miniCBEs. To this end, mutated rAPOBEC1 (W90Y and R126E) [39], Anc689 APOBEC1 [12], or mutated hA3A (R130F or W98Y/W104A/Y130F) [70,71] was, respectively, fused to dUn1Cas12f1 while varying the position [69]. As in the miniABEs, each miniCBE exhibited different editing scopes with various editing efficiencies. In addition, the editing efficiency was further enhanced by mutating Un1Cas12f1 (D143R/T147R/E151A, RRA). Notably, the enhancement of the editing efficiency was accompanied by a broadening of the editing window. To achieve efficient editing with high precision, reprogrammed TadA [72,73,74] was adopted instead of conventional cytidine deaminases [69]. The CBE, named d12fCBEs-8e, was further improved by introducing mutations (V28G/A48G/I49A/V82T/N108Y and GGATY). The resulting d12fCBEs-8e (GGATY) exhibited efficient C-to-T editing with minimal A-to-G editing at single-nucleotide resolution. Furthermore, the off-target effects on the DNA and RNA were low. Both miniABE [N-dRRAABE-TadA* (82G)] and one d12fCBE-8e (GGATY) variant [NdRRACBE-8e (GGATY)] were also effective for correcting pathogenic mutations in the *CLCNKB* and *VHL* genes of HEK293T cells. In particular, N-dRRACBE-8e (GGATY) also induced C-to-T editing in the *Polg* (26.1%) and *Foxp1* (25.8%) genes with minimal off-target effects when delivered to a mouse brain via AAV vectors.

In a recent study, a different combination of mutations (D143R/T147R/T203R/E206R) was introduced to enhance the activity of Un1Cas12f1 (Un1Cas12f1QM) [75]. When dUn1Cas12f1QM was adopted in ABE7.10, the resulting UminiABE outperformed the miniABE exploiting dCasMINI. To further improve the UminiABE, a small DNA-binding protein, Sso7d from *Sulfolobus solftaricus* [76,77,78], was fused to the UminiABE, and the nonessential regions in the gRNA were deleted (STUminiABE) [75]. The STUminiABE exhibited A-to-G editing with an efficiency ranging from 35.75% to 62.24%. For C-to-T editing, the adenine deaminase of the STUminiABE was replaced with mutated APOBEC3A, and UGI was added (STUminiCBE). The editing efficiency of the STUminiCBE ranged from 25.33% to 64.80% in the genomic sites of HEK293FT cells. The STUminiCBE is small enough to be loaded into a single AAV vector, even though it includes an additional DNA-binding domain (Sso7d). The application of STUminiCBE using an AAV vector for C-to-T editing resulted in an efficiency of 34.02%. Despite the performance and compatibility of STUminiCBE and STUminiABE with a single AAV system, high off-target effects remain a problem.

CWCas12f originates from the *Candidatus woesearchaeota* archaeon and is very similar to Un1Cas12f1 in its sequence [79]. This similarity suggests that both Cas proteins may share properties. Like Un1Cas12f1, CWCas12f shows little activity with the canonical gRNA in eukaryotic cells. Instead, CWCas12f becomes effective with engineered gRNA. CWCas12f with engineered gRNA was termed the tiny nuclease/augment RNA-based genome editing technology (TaRGET) and combined with a codon-optimized TadA-TadA* (V106W/D108Q) heterodimer to produce a novel small ABE (TaRGET-ABE-C3.0). Although TaRGET-ABE-C3.0 exhibited higher editing activity compared with dCasMINI-ABE, it was inferior to that of the SpCas9-based ABEs, such as ABE7.10, ABE8e, ABE9, and STUminiABE [9,15,29,67,75]. The size of TaRGET-ABE-C3.0 is so small that additional gRNAs can be loaded for multiplex editing. As predicted, an AAV carrying Target-ABE-C3.0 with paired gRNAs carried out A-to-G editing at two target sites without compromising the activity of HEK293T cells. Because CWCas12f recognizes TTTR PAM, just like Un1Cas12f1, the targetability of TaRGET-ABE-C3.0 was restricted. To overcome this limitation, diverse mutations were introduced into dCWCas12f in TaRGET-ABE-C3.0 to generate TaRGET-ABE-C3.0 variants that recognize different PAM sequences. The editing window of TaRGET-ABE-C3.0 was broadened by introducing an additional mutation (I159W) or by adopting TadA-8e from ABE8e with the wild-type TadA as a dimer (TaRGET-ABE-C3.1). When an AAV harboring TaRGET-ABE-C3.1 was injected into a mouse tail, the highest editing efficiency (10.9%) was observed in the liver. A-to-G editing was also observed in the heart, muscle, and testis, although the efficiency did not reach that in the liver. TaRGET-ABE-C3.1 also exhibited detectable off-target effects. In particular, the RNA off-target effect of TaRGET-ABE-C3.1 was higher compared with that of TaRGET-ABE-C3.0, indicating the necessity of additional optimization.

MmCas12m is another small Cas12 variant (596 amino acids) that originates from *Mycolicibacterium mucogenicum* CCH10-A2 [80,81]. Similar to other Cas12 orthologs, Cas12m has a PAM preference at the T-rich motif (TTN) [80]. To generate a small CBE (dCas12m-CBE1), dCas12m was fused to the PmCDA1-UGI complex [14]. Interestingly, Cas12m cannot cleave dsDNA, although its DNA-binding activity is intact. Consistent with this, Cas12m-CBE1 and dCas12m-CBE1 exhibited similar editing activity. For dCas12m-CBE2 and dCas12m-CBE3, the codons of PmCDA1 and UGI were optimized with the *Homo sapiens* codon. Both dCas12m-CBE2 and dCas12m-CBE3 showed lower editing efficiency compared with dCas12m-CBE1 because the codon optimization may have reduced protein synthesis in *Escherichia coli*. Unfortunately, the Cas12m-based BEs have not been tested in human cells.

GoCas12m from *Gordonia otitidis* is slightly different from MmCas12m. GoCas12m consists of 607 amino acids and recognizes the TC-rich motif as a PAM sequence [82]. Like MmCas12m, GoCas12m binds to dsDNA in a gRNA-dependent manner, but does not cleave it. GoABE is a fusion protein of GoCas12m and TadA-8e [15,82]. GoABE induced A-to-G editing in HEK293T cells with a 19% efficiency, which is comparable to that of enAsABE composed of engineered AsCas12 and TadA-8e, whereas the editing windows were different [15,82,83].

Recently, a family of 10 Cas12j (CasΦ) nucleases was identified, and their sizes ranged from 700 to 800 amino acids [84]. In a subsequent study, six Cas12j orthologs were tested as gene editing tools, with Cas12j-8 showing the highest activity [85]. Cas12j-8 recognized TTN PAM preferentially and exhibited higher specificity compared with Cas12a from *Francisella novicida* [85,86]. For base editing, dead Cas12j-8 (dCas12j-8) was fused to TadA-8e [15] and Cas12j-8ABE8e carried out A-to-G editing at some target sites with various efficiencies (5.1–11.3%) in HEK293T cells [85]. However, the editing efficiency was too low to be detected when Cas12j-8ABE8e was delivered via an AAV vector.

## 5. Harnessing the Ancestors of Cas Proteins

As phylogenetic research has expanded, the ancestral systems of the CRISPR-Cas system have been identified (Figure 3C), which have been grouped as the obligate mobile element-guided activity system (OMEGA system) [63,87,88]. In the IS200/IS605 superfamily of transposons, the insertion sequence Cas9-like OrfB (IscB) has an RuvC domain and an HNH domain, similar to Cas9, but smaller in size (496 amino acids), which is supposed to be an ancestor of the Cas9 protein [87,89]. Like Cas9, IscB cleaves DNA in an RNA-guided manner (ωRNA), with a preference for a specific target-adjacent motif (TAM) sequence [89]. However, the activity of native IscB was too low in HEK293T cells (up to 2%) to be used as BEs. To enhance its activity, ωRNA was truncated and mutated (ωRNA*), and IscB from the human gut metagenome (OgeuIscB) that recognizes NWRRNA as a TAM was engineered (E85R/H369R/S387R/S457R) (IscB*), generating an enhanced IscB system (enIscB) [90]. To generate an ABE, TadA-8e (V106W) [15] was fused to enIscB nickase at both terminals and the IscB-based ABE, designated miABE, exhibited A-to-G editing with an efficiency of 52.37% and 60.06% at the two loci. For C-to-T editing, mutated hA3A (W104A) [71] was fused to enIscB nickase (miCBE) [90]. The miCBE showed editing efficiencies of 66.42% and 50.17% at the two target sites. Compared with the SpCas9-based BEs, both miABE and miCBE exhibited better nuclease-independent off-target effects.

In another study, a different type of IscB mutation (D97K/F138N/S431K/S457R) and ωRNA optimization were introduced to enhance OgeuIscB activity [91]. By fusing a mutated APOBEC3A (W104A/Y132D) [71] to the engineered IscB nickase (F138N/S431K), IminiCBE was produced. It accomplished C-to-T editing with an efficiency ranging from 18.68% to 92.61% in HEK293FT cells [91]. Likewise, IminiABE with a TadA-TadA-8e heterodimer exhibited A-to-G editing with efficiencies ranging from 21.53% to 92.03%. This group also developed IminiCGBE and IminiAYBE. IminiCGBE3 that harbored eA3A showed diverse C-to-G editing at various efficiencies depending on the target site, whereas the purity was >40%. For IminiAYBE, an N-methylpurine DNA glycosylase mutant (MPGm) [92] was used. Its A-to-Y editing efficiency was less than 20% with various purities. Adding the DNA-binding-domain Sso7d enhanced the performance of the IminiBEs (SIminiBEs). Because the IminiBEs and SIminiBEs exhibited a near-TAM-less requirement (NNRR > NNRY > NNYY), it was expected that they would have more potential off-target sites. However, the off-target effects of the IminiBEs and SIminiBEs were just comparable to those of the SspCas9-based BEs.

At the same time, the activities of several IscB orthologs were tested [93]. Of 19 IscB orthologs, IscB.m16 exhibited the highest activity. Similar to other studies, the performance of IscB.m16 was enhanced through ωRNA engineering (enωRNA) and mutating IscB.m16 (E326R, P460S, T462H, and T459E) (IscB.m16*). Notably, IscB.m16* recognized NNNGNA TAM, whereas the wild-type IscB.m16 with natural ωRNA recognized MRNRAA (M = A or C) TAM. IscB.m16* was also adopted to produce BEs. To this end, mutated hA3A (W104A) and TadA-8e (V106W) were fused to IscB.m16* to produce IscB.m16*-CBE and IscB.m16*-ABE, respectively [15,71,93]. The editing efficiency of IscB.m16*-CBE (60.01%) in HEK293T cells was comparable to that of the engineered OgeuIscB-CBE (enOgeuIscB-CBE) and SpCas9-CBE. Interestingly, the editing efficiency of IscB.m16*-ABE (46.15%) was higher compared with that of enOgeuIscB-ABE, and comparable to that of SpCas9-ABE. The off-target effects (gRNA dependent and gRNA independent) of IscB.m16*-ABE were comparable with those of enOgeuIscB-ABE and SpCas9-based ABE. As expected, IscB.m16*-CBE could be loaded into a single AAV vector. When the AAV vector that carried IscB.m16*-CBE targeting the dystrophin gene was injected into a Duchenne muscular dystrophy mice model, there was a G-to-H (H = A, T, or C) conversion with an efficiency of 7%, along with a 40% increase in the dystrophin protein levels.

In addition to IscB, the IS200/IS605 transposon superfamily includes the RuvC-like endonuclease TnpB, which is supposed to be the ancestor of Cas12a [63]. IsDra2 TnpB from *Deinococcus radiodurans* consists of 408 amino acids and exhibits DNA cleavage activity with a TTGAT TAM preference in human cells [94]. Similar to IscB, TnpB forms an RNP complex with the right-end element (reRNA) of the transposon, and the reRNA guides TnpB to the target site. For base editing in plants, codon-optimized TnpB was catalytically deactivated and fused to TadA-8e at either the N- or C-terminus [15,95]. dTnpB-ABE8e-N and dTnpB-ABE8e-C performed A-to-G editing with efficiencies ranging from 0.42% to 1.12% [95].

## 6. Employment of Small Deaminases

In recent studies, deaminases were classified with the assistance of artificial intelligence (AI) [96,97,98]. The clade of SCP1.201 deaminases was shown to include small cytidine deaminases that can interact with ssDNA or dsDNA, respectively [99]. Through the characterization of ssDNA cytidine deaminases in HEK293T cells, referred to as ssDNA deaminases (Sdds), a higher base editing efficiency was observed for Sdd7, Sdd9, Sdd5, Sdd6, Sdd4, Sdd76, and Sdd10 (in order of editing efficiency), compared with that for rAPOBEC1-based CBE. Notably, Sdd7, Sdd9, and Sdd6 did not show sequence preferences, whereas Sdd3 preferred the GC and AC motifs. In addition, the off-target effects of Sdd2, Sdd3, Sdd4, Sdd6, Sdd10, and Sdd59 were lower compared with that of rAPOBEC1 in rice protoplasts. In HEK293T cells, the editing efficiency of Sdd6 was comparable to that of the rAPOBEC1-based CBE, and the ratio of on- and off-target editing was superior to that of high-fidelity CBEs, such as YE1 and YEE [99,100]. Using AI, Sdds were truncated to a size of 130–160 amino acids (mini-Sdd) [99]. Along with SaCas9, the mini-Sdd6 constituted a CBE, which could be loaded into a single AAV vector. The application of an AAV vector carrying mini-Sdd6-based CBE to mouse neuroblastoma N2a cells exhibited an editing efficiency of up to 43.1%, which was dependent upon the AAV concentration.

In an independent study, several Sdd orthologs were identified in a similar manner [101]. To validate the editing ability, each Sdd ortholog was adopted in AncBE4max [12], and SflSdd-CBE 1.0 and Air1Sdd-CBE 1.0 showed an editing efficiency comparable to that of AncBE4max. SviSdd-CBE 1.0 exhibited a moderate editing efficiency in HEK293T and HeLa cells [101]. Considering the delivery of Sdd-CBE via a AAV vector, the three Sdds were truncated to reduce their size (Sdd-CBE 2.0). Interestingly, the truncation enhanced the activity of SflSdd-CBE 2.0 and SviSdd-CBE 2.0 at some target sites. The activity of Sdd-CBE 2.0 was further increased by adding a short viral element (ek5) (Sdd-CBE 3.0) [102], which stabilized the mRNA and enhanced translation, thus improving the editing efficiency. Perhaps because of the enhanced activity, Sdd-CBE 2.0 and Sdd-CBE 3.0 showed slightly higher byproducts (G-to-A, C-to-G, and C-to-A editing) compared with their original constructs. In addition, off-target effects were also induced by the Sdd-CBEs. Nevertheless, the non-C-to-T mutations and DNA off-target effects were lower with SflSdd-CBE 3.0 and SviSdd-CBE 3.0 compared to those with AncBE4max.

To load SflSdd-CBE 2.0 into a single AAV vector, nCas9 was replaced with nSaCas9, and the UGI was removed to reduce the size to 4505 bp. The injection of an AAV vector carrying a small CBE, known as SflSdd-CBEs (nSaCas9) 2.0, targeting the *PCSK9* gene in mice, showed C-to-T editing with an efficiency of 15% and a reduction in cholesterol levels to 78%. No signs of liver damage by the AAV were observed.

## 7. Adoption of the Type I-F CRISPR-Associated Complex for Antiviral Defense (Cascade)

**Figure 4:**
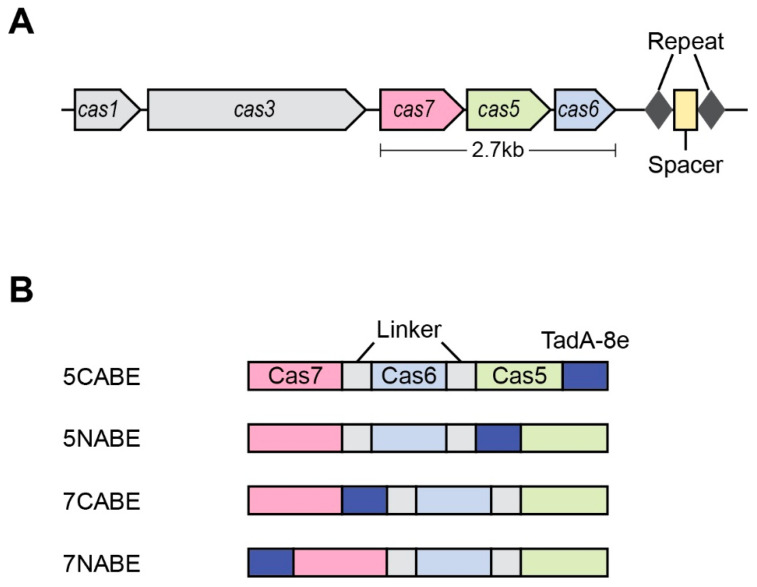
Type I-F2 cascade system for producing ABEs. (**A**) Schematic diagram of the *Moraxella osloensis* strain CCUG 350 type I-F2 CRISPR-Cas system. (**B**) ABE variants are based on the type I-F2 cascade.

Although the CRISPR systems belonging to type II (Cas9) and V (Cas12) have been actively exploited for gene editing, the application of the type I system is challenging because of its complex composition. A recent study focused on the type I-F2 system derived from *Moraxella osloensis* CCUG 350 (Mos350), which is a compact system (2.7 kb) composed of Cas1, Cas3, Cas5, Cas6, and Cas7, but lacking a large and small subunit (Figure 4A) [103], consistent with previous studies [104,105,106]. To exploit the Mos350 I-F2 system for gene editing in human cells, a nuclear localization signal was added to the human codon-optimized Cas5-Cas6-Cas7 Cascade [103]. In addition, the transcription of crRNA was enhanced by changing the spacer length, and the DNA-binding activity of the Mos350 Cascade was improved by introducing mutations (L175F) into the Cas7 protein. When the engineered Mos350 Cascade system was tested using VPR, the activity was enhanced by 16.7-fold and 5.3-fold at two target sites with a CC PAM preference in human cells. No off-target effects were observed. For adenine base editing, TadA-8e was fused to either the C- or N-terminus of Cas5 or Cas 7 in the Mos350 Cascade (L175F) to generate 5CABE, 5NABE, 7CABE, and 7NABE, respectively (Figure 4B) [15,103]. Although the four Mos350 Cascade-based ABEs exhibited diverse editing efficiencies depending on the target sites, the editing windows of the ABEs were usually wide, which resulted from the wider R-loop formation compared with that of Cas9 or Cas12 [107,108,109]. Interestingly, the editing efficiencies of each adenine formed a bimodal distribution across the editing window and the position of the best editing adenine was different depending on the ABE variant. Of the four ABE variants, 5NABE showed the highest editing efficiency (30.4%) at position 27. 5NABE also achieved A-to-G editing at the target sites containing a single adenine within the editing window with efficiencies ranging from 7.4% to 24.3%. Notably, the editing of some adenines positioned outside of the protospacer was also observed. Although 5NABE exhibited Cas-dependent off-target effects related to the expression level of the ABE system, no significant Cas-independent off-target effects were detected.

## 8. Efforts to Overcome Other Limitations of BEs

### 8.1. Off-Target Effects

The off-target effects are a common challenge of gene editing tools, including BEs, and can be fatal in clinical applications. In BEs, the off-target effects are primarily Cas-independent, suggesting that the activity of the deaminase is the main cause of non-specific editing. In many studies, the deaminase activity at the off-target sites has been regulated by introducing mutations into the deaminase [15,30,100,110,111,112,113].

Alternatively, the BE activity can be regulated by splitting the deaminase (split-BE), which can be reassembled through chemically induced protein dimerization [114]. Each fragment of the split deaminase is fused with either the FK506 binding protein (FKBP) or the FKBP-rapamycin binding domain (FRB). In the presence of rapamycin, FKBP and FRB combine, resulting in the reconstitution of the split-BE [115,116,117]. A more precise control of the split-BE can be achieved by using photoswitches named Magnets, which consist of pMag and nMag, small photoreceptors that dimerize in response to blue light [118]. This allows for the activity of the split-BE to be spatiotemporally regulated by blue light (BLBE) [119]. However, the phototoxicity of blue light presents a challenge for in vivo applications of BLBE. A recent study demonstrated that near-infrared (NIR) light can induce the reassembly of split-BE using upconversion nanoparticles, which convert NIR into blue light [120]. With the lower phototoxicity and higher tissue penetration of NIR, the NIR-activated BE successfully conducted base editing in the liver of mice.

Since steric hindrance is supposed to influence the off-target effects of BE [70], embedding the deaminase within the Cas protein (inlaid BE) could be another strategy for reducing the off-target effects. Consistent with this idea, an inlaid BE has demonstrated lower off-target effects in several studies [30,43,72,121]. Notably, embedding the deaminase within the Cas protein has resulted in the widening or shifting of the editing window [122,123,124].

The activity of the deaminase in a BE can also be regulated using its inhibitor. In the transformer BE (tBE) system, a cleavable deoxycytidine deaminase inhibitor (dCDI) is conjugated with a BE to suppress its activity [125]. When the tBE binds to the on-target site, the dCDI is removed by TEV peptidases recruited by the gRNA. As a result, the tBE becomes active only at the on-target site, minimizing the off-target effects.

Repurposing the deaminase is another strategy to reduce the off-target effects of BEs. Several studies have reported that ABEs exhibit lower off-target effects than CBEs and mediate C-to-T editing as well as A-to-G editing [73,126,127]. Based on this, novel CBEs employing engineered TadA with reduced off-target effects have been developed [69,74,128,129,130,131,132].

### 8.2. Bystander Mutations

Although BEs can precisely edit genes at the single-nucleotide level, they are susceptible to bystander mutations when multiple cytosines or adenines are present within the editing window. In clinical applications of BEs, these unintended mutations can be detrimental. In addition, the presence of bystander nucleotides may interfere with the efficiency of the target nucleotide editing [133,134,135]. One way to reduce bystander mutations has been to narrow the editing window through the engineering of deaminase or linkers [13,39,57,111,136,137,138,139,140]. While these attempts have reduced the size of the editing window to approximately two nucleotides, one recent study successfully developed a CBE with single-nucleotide resolution [141]. Alternatively, enhancing the preference for a specific sequence motif could help reduce bystander mutations. Engineering the deaminase has allowed CBEs and ABEs to function with a preference for the TCR or TCCR motif and the TA or CA motif, respectively [142,143]. As a result, the bystander cytosines or adenines that were not part of these motifs could remain unaffected by the BEs.

### 8.3. Limited Editing Scopes

Because existing deaminases can only mediate base transitions, CBEs and ABEs are limited in transversion editing. However, 42% of human pathogenic single-nucleotide polymorphisms result from transversion mutations [92], raising the need for novel BEs capable of inducing transversion editing. Most CBEs contain UGI to inhibit DNA glycosylase, as this inhibition enhances C-to-T editing [8]. Conversely, activating DNA glycosylase may enable C-to-A or C-to-G editing. Consistent with this idea, several studies have shown that BEs having DNA glycosylase instead of UGI, such as GBE, CGBE, and OPTI-CGBE, can mediate C-to-A (with AID) or C-to-G (with rAPOBEC1) editing [144,145,146,147]. Since C-to-A/G conversion is mediated by the base excision repair (BER) pathway, fusing a BER-associated protein, such as X-ray repair cross-complementing protein 1 (XRCC1), with CBEs can also facilitate C-to-G editing [148]. Likewise, ABEs with DNA glycosylase have exhibited A-to-C or A-to-T transversion editing (ACBE or AYBE) [92,149].

There have also been attempts to develop novel BEs that can directly edit G or T rather than C or A. Because these BEs do not require cytidine or adenosine deamination, they utilize DNA glycosylase without deaminases. In one study, a deaminase-free, glycosylase-based guanine BE (gGBE) was created by fusing mutated DNA glycosylase to the Cas9 protein, which mediated G-to-Y editing [150]. In a similar way, novel glycosylase-based BEs have been developed, which mediated T-to-S (gTBE) and C-to-G editing (gCBE), depending on the mutation types in the DNA glycosylase [151].

Although BEs typically target a single type of nucleotide, heterogeneous base editing could expand their range of applications. To achieve this, BEs capable of catalyzing both C-to-T and A-to-G edits simultaneously have been developed by combining a cytidine deaminase and an adenosine deaminase, such as A&C-BEmax (ABE7.10 + AID-BEmax), SPACE (miniABEmax-V82G + Target-AID), Target-ACEmax (Target-AID + ABE7.10), ACBE (Target-AID + ABE7.10), iACBE (evoCDA1 + ABE9e), and STEMEs (APOBEC3A + ecTadA) [42,152,153,154,155,156]. In a recent study, CGBE and ABE were combined to produce AGBEs that could install four types of conversions (C to G, C to T, C to A, and A to G) with various editing efficiencies [157].

Despite the versatility of BEs, editing organelle DNA, such as mitochondrial DNA (mtDNA), remains a significant challenge due to the lack of a method for delivering gRNA into mitochondria [158]. To address this issue, the CRISPR system was replaced with a transcription activation-like effector (TALE) and an interbacterial toxin (DddA) was employed as the cytidine deaminase, leading to the development of DdCBE, a gRNA-free BE. DdCBE can be delivered into mitochondria by a mitochondrial-targeting signal and recruited to the target site by a TALE array. While DddA was split to minimize its toxicity in the initial version of DdCBE, a later study employed a full-length DddA with reduced toxicity achieved by mutations [159]. Additionally, DdCBE was modified into TALED, which enabled adenosine deamination by incorporating engineered TadA without UGI [160]. More recently, a study introduced crDdCBE, which combined DddA with the CRISPR system for nuclear DNA editing [161].

## 9. Conclusions

BEs are undoubtedly one of the most innovative gene editing technologies based on the CRISPR system, and the superiority of BEs is evident for potential clinical applications. A single-nucleotide mutation can lead to the generation of functionally aberrant proteins that cause genetic diseases (>58% of the entries in the ClinVar database) [162]. Gene editing at single-nucleotide resolution using BEs is ideal for treating such diseases.

The safe and effective delivery of gene editing tools is an important factor in determining the efficiency of gene editing. Although AAV vectors have been in the spotlight as a practical delivery system, loading BEs into AAV vectors is limited because of their sizes, which are close to or exceed the capacity of AAV vectors. Downsizing the BEs has emerged as a solution, which includes truncating the components of the BEs and employing smaller variants of the Cas protein and deaminase (Table 1). In addition, diverse efforts are underway to address other issues, such as off-target effects, bystander mutations, and the limited editing scope.

The clinical application of BEs still has a long way to go. However, with continuous advancements and their unique editing abilities, BEs will become a powerful tool, driving the further expansion of their applications in the future.

## Figures and Tables

**Table 1:** A list of small-sized BEs and strategies for downsizing.

Name of BE	Strategy for Downsizing	Approximate Size (Excluding the Promoter)	Ref.
sABE	Dual deletion of REC2 and HNH	~4.15 kb	[27]
SaAID-2S	Truncation of PmCDA1 and adoption of SaCas9	~3.9 kb	[30]
SaAID-3S		~3.9 kb	
SaBE3	Adoption of SaCas9	~4.2 kb	[39]
SaBE3-KKH		~4.2 kb	
SaKKH-ABE7.10		~4.3 kb	[40]
SaKKH-ABE8e		~3.9 kb	[15]
miniABEmax (V82G)-nSaCas9		~3.9 kb	[43]
miniABEmax	Removal of wtTadA	~4.8 kb	[42]
SauriBE4max	Adoption of SauriCas9	~4.5 kb	[44]
SauriABEmax		~4.6 kb	
SauriABE8e		~3.9 kb	[45]
cjABE8e	Adoption of CjCas9	~3.6 kb	[45]
cjCBEmax		~4.3 kb	[51]
nNme2-CBE	Adoption of Nme2Cas9	~4.6 kb	[55]
Nme2-ABE8e		~3.9 kb	[45,59]
nNc-CBE	Adoption of NcCas9	~3.9 kb	[60]
nNc-ABE		~3.9 kb	
dCasMINI-ABE	Adoption of Un1Cas12f1	~3.0 kb	[67]
miniABE		~2.3 kb	[69]
miniCBE		~2.5 kb	
UminiABE		~2.6 kb	[75]
STUminiABE		~2.8 kb	
STUminiCBE		~2.4 kb	
TaRGET-ABE-C3.0	Adoption of CWCas9	~3.0 kb	[79]
TaRGET-ABE-C3.1		~3.0 kb	
dCas12m-CBE1	Adoption of MmdCas12m	~3.1 kb	[80]
GoABE	Adoption of GoCas12m	~2.8 kb	[85]
miABE	Adoption of enIscB	~2.8 kb	[90]
miCBE		~2.8 kb	
IminiCBE	Adoption of OgeuIscB	~2.4 kb	[91]
IminiABE		~2.6 kb	
IminiCGBE		~2.4 kb	
IminiAYBE		~3.5 kb	
SIminiBE (CBE)		~2.6 kb	
SIminiBE (ABE)		~2.8 kb	
enOgeuIscB-CBE		~2.4 kb	[93]
enOgeuIscB-ABE		~2.6 kb	
dTnpB-ABE8e-N	Adoption of ISDra2 TnpB	~2.0 kb	[95]
dTnpB-ABE8e-C		~2.0 kb	
mini-Sdd6	Adoption of Sdd6	~4.0 kb	[99]
SflSdd-CBE	Adoption of SflSdd	~4.5 kb	[101]
5CABE	Adoption of type I CRISPR	~3.4 kb	[103]
5NABE		~3.4 kb	
7CABE		~3.4 kb	
7NABE		~3.4 kb	
